# The Value of Ultrasound Monitoring of Adnexal Masses for Early Detection of Ovarian Cancer

**DOI:** 10.3389/fonc.2016.00025

**Published:** 2016-02-10

**Authors:** Elizabeth Suh-Burgmann, Walter Kinney

**Affiliations:** ^1^Division of Gynecologic Oncology, The Permanente Medical Group, Walnut Creek, CA, USA

**Keywords:** ultrasound detection, adnexal diseases, ovarian neoplasms, early detection of cancer, overtreatment

## Abstract

Although ultrasound has so far been found to be ineffective as a screening tool for ovarian cancer, it is commonly used as a means of evaluating or following ovarian or adnexal masses once they are detected. We review the use of serial ultrasound for the management of adnexal masses and propose an approach to monitoring based on an understanding of the overall risk of cancer among the population in question and an assessment of how the potential benefit of monitoring compares with potential risk. In our approach, masses that are symptomatic, large (>10 cm), associated with an elevated CA 125 level or overt signs of malignancy, or that are determined to have a worrisome appearance by stringent ultrasound criteria should be evaluated surgically. Women with masses that have none of these characteristics should be offered monitoring. Short-term initial ultrasound monitoring carries significant potential benefit in terms of aiding detection of early malignancy and avoidance of unnecessary surgery. However, if a mass remains stable but persistent, the potential benefit of ongoing monitoring wanes with time, whereas the potential harms, in terms of patient anxiety, cost, and the risk of incidental findings and unnecessary surgery increase. Therefore, monitoring of stable lesions should be limited in duration in order to limit potential harms from overtreatment and overdiagnosis.

## Introduction

Although the majority of women with epithelial ovarian cancer present with late stage disease, approximately 15% have early stage disease at diagnosis (1). Many early stage cases present with a large mass or worrisome clinical signs, but for a small subset, the initial presentation is a small, asymptomatic adnexal mass with no other factors that would raise suspicion of cancer. Biopsy of adnexal masses is generally not recommended since ovarian cancer is known to spread by direct peritoneal extension, and therefore, if a mass is malignant, biopsy could theoretically worsen prognosis. As a result, concern that a mass in an older woman may represent an early cancer leads many women with small masses to undergo unnecessary surgery with accompanying morbidity, despite the fact that the overwhelming majority of these masses are found to be benign.

The alternative to immediate surgery for masses of uncertain nature is ultrasound monitoring. Here, we discuss when monitoring of masses in postmenopausal women should be considered, the distinction between initial short-term ultrasound monitoring and prolonged monitoring in terms of potential value, as well as potential harms. Based on these considerations, we propose an approach to ultrasound monitoring for adnexal masses based on published clinical data that aims to maximize benefit and minimize harm.

## When Should Monitoring be Considered?

Surgery is appropriate for symptomatic masses and masses that are associated with other signs of malignancy, such as elevated CA125 (in postmenopause), ascites, or evidence of metastatic disease, or in women at high genetic risk for ovarian cancer. Surgery is also appropriate for large masses (>10 cm), which are less likely to regress, have a higher risk of symptoms, and are often more difficult to characterize on ultrasound. Therefore, the women for whom ultrasound monitoring is an option are women whose presentation does not include any of these characteristics: average risk women with smaller, asymptomatic masses.

## What is the Risk of Cancer?

Appropriate management of women with smaller asymptomatic masses should be based on the risk of cancer – the lower the risk of cancer is for a group, the lower the rate of surgery should be. Unfortunately, there is little “real world” data on what the risk of cancer is among women who are potential candidates for monitoring. The traditional teaching within gynecology has been that complex adnexal masses in older women are cancer until proven otherwise. However, this view was not based on the subset of women with characteristics that would make them candidates for observation, and it was often drawn from referral populations where the prevalence of cancer is elevated (2–5). Furthermore, this impression of risk was established during an era when most masses came to the attention of patients and providers due to symptoms or being palpable on exam. It is clear that with rising rates of utilization of imaging of all types, including office ultrasound, adnexal masses are increasingly found incidentally in studies obtained for an entirely separate concern (6–8). Trial data demonstrate that among older women who have an adnexal mass identified through ultrasound screening, the overall risk of invasive cancer is approximately 1–2% (9–11). In the UKCTOCS trial, an ongoing randomized controlled screening trial of over 20,000 postmenopausal women in the United Kingdom, of 48,230 women who had an initial ultrasound screen, 9.1% had an abnormal scan, and among these women, the absolute risk of epithelial ovarian cancer over the following 3 years was 1.08% (9). Although the risk of cancer for women identified by screening is expected to be lower than the risk for women found to have a mass in clinical practice, the degree to which they differ will depend on the proportion of women in clinical practice who are diagnosed with a mass as the result of signs or symptoms related to ovarian malignancy. One in every three physicians reportedly engage in ovarian cancer screening of low-risk women despite evidence to date that ovarian cancer screening using transvaginal ultrasound and CA125 tumor marker testing is ineffective at reducing ovarian cancer mortality (12). In addition to inappropriate screening, the widespread use of ultrasound effectively results in inadvertent screening. Unlike mammography, which is rarely used for any indication other than screening, pelvic ultrasound is used for a range of clinical indications, such as checking IUD placement or evaluating fibroids. If the UKCTOCS experience holds, each ultrasound exam on a postmenopausal woman has a 9% chance of incidentally finding an adnexal mass. Although large masses or masses associated with ascites are usually diagnosed as a result of symptoms, the women who are candidates for ultrasound monitoring are women with small isolated asymptomatic masses. For these reasons, the difference in the risk of malignancy observed for women identified by screening compared to women who undergo monitoring may not be as great as expected. We evaluated the risk among women who were found to have small masses in the course of routine care by identifying a population-based cohort of 1363 women over age 50 with complex masses <6 cm in size not associated with other evidence of cancer (13). A total of 7 cancers and 11 borderline tumors were found with 24 months of follow-up. The majority (994/1363, 73%) underwent ultrasound monitoring, with 5 of the cancers and 7 of the borderline tumors found in the monitored group during follow-up for an overall risk of 1.3%, and 0.5% for invasive cancer specifically.

## Triage to Surgery Versus Observation

Several strategies have been proposed to better identify masses that are likely to be malignant. Although elevated CA125 levels raise the likelihood of cancer, a normal value is seen in approximately 50% of early stage cases (14), and therefore, does not exclude possible cancer. Whether longitudinal measurement of CA125 over time, evaluated by the risk of ovarian cancer algorithm (ROCA), will be effective as a screening method for low-risk women is a question currently being studied within the multimodality screening arm of UKCTOCS (15). A number of algorithms that combine clinical and ultrasound criteria have been proposed. The risk of malignancy index (RMI) is a score generated by assessment of ultrasound features, menopausal status, and the serum CA125 level (international unit per milliliter). The ultrasound features in RMI are multilocularity, solid areas, and bilaterality (16). The International Ovarian Tumor Analysis (IOTA) group developed two logistic regression models (LR1 and LR2), which rely more heavily on ultrasound features and also include age, personal history of ovarian cancer, and tenderness of the mass on physical exam (LR1) to predict malignancy (17). The ultrasound findings that lead to a higher score are ascites, blood flow in papillary projections, solid nature of the tumor, maximum diameter of the largest solid component, irregular internal cyst wall, lack of acoustic shadows, and higher degrees of vascular flow. The group also developed and evaluated a set of “simple rules (SR)” that produce a score based solely on the presence or absence of benign or malignant ultrasound features, in which malignant features are defined as irregular solid tumor, ascites, at least four papillary projections, irregular multilocular solid tumor at least 10 cm, and very strong intratumoral blood flow (18). Recently, they reported an analysis of their studies in which they found that all IOTA strategies (LR1, LR2, SR, and combinations of the above) were superior to RMI for predicting malignancy among masses with sensitivities in the range of 90–96% and specificity of 74–79% (19). Interestingly, they found LR1 was only slightly more sensitive but significantly less specific than “subjective assessment” alone which relied entirely on expert radiology impression (93.7, 77.6 versus 92.5, 87.7, respectively). However, the generalizability of these findings is debatable due to a higher prevalence of cancer in the populations studied as well as a level of radiology expertise that may not be reproducible in other settings (20). Finally, investigators from the Kentucky ovarian screening study developed a “morphology index” (MI) based on mass volume and proportion of solid component, and found that in their study, 85% of cancers and borderline tumors had a score of at least 5 at the time that the decision was made for surgery (21). Although debate continues regarding the superiority as well as generalizability of one strategy compared to another, from a practical standpoint, clinical criteria, such as personal history of ovarian cancer, elevated CA125, and evidence of ascites or metastases, as well as large mass size >10 cm, are already generally considered sufficient reason to direct a woman with a mass to immediate surgical evaluation. Therefore, further triage of women without these characteristics to either ultrasound observation versus surgery relies mainly on ultrasound characteristics. Among the ultrasound features that are associated with malignancy, the presence of large solid areas is the most consistent characteristic included in ultrasound-based prediction strategies. The significance of solid areas has also been demonstrated in screening trials. In UKCTOCS, masses without solid elements had an absolute risk of 0.4%, whereas masses with solid elements had an absolute risk of 4.45% (9). Analysis of the ultrasound abnormalities seen in PLCO also found that both the size of the mass and the presence of solid components correlated with risk of malignancy, with all masses <5 cm and larger masses without solid areas being low risk (22). Requiring solid components to demonstrate vascular flow by Doppler has been shown to increase the specificity of morphology for malignancy (23–26). Given the overall low risk of malignancy among women who are candidates for monitoring, the ultrasound criteria used to exclude women from initial short-term monitoring should be highly specific, in order to avoid exposing women to excessive unnecessary surgery. In our practice, we support excluding only masses that demonstrate significant solid vascular components from consideration of initial monitoring.

## Schedule of Monitoring

When considering ultrasound monitoring, a distinction must be made between initial, short-term repeat exam, limited monitoring for up to 1–2 years and indefinite, potentially life-long monitoring of stable masses. Initially, monitoring serves to identify masses with aggressive growth patterns, and it helps to avoid surgery on masses that are benign or transient in nature such as hemorrhagic cysts. The Society of Radiologists in Ultrasound published guidelines in 2010, based on committee consensus opinion, which recommended a follow-up interval of “6–12 weeks” for indeterminate masses among premenopausal or perimenopausal women, but immediate surgical consideration for postmenopausal women (27). However, there is growing consensus that a repeat exam in 6–8 weeks is safe and does not negatively impact stage at diagnosis (28–30). In the Kentucky ovarian cancer screening study which used serial transvaginal ultrasound as well as CA125, it was found that over 75% of cystic and solid lesions resolved on monitoring over 12 months (28). The investigators credit the use of serial ultrasound in decreasing the rate of false positive results and did not find that initial monitoring resulted in more advanced stage at diagnosis. A similar strategy is used in the ultrasound only arm of the UKCTOCS trial in which women with initial ultrasound abnormalities are directed to undergo a repeat ultrasound 6–8 weeks later that is performed by a more experienced ultrasonographer (30). Only if the mass is persistent at that time is a clinical assessment made regarding the suspicion for cancer. Indeterminate masses that are stable on the initial 6- to 8-week exam can be further monitored. The American College of Obstetrics and Gynecology (ACOG) Practice Bulletin on Management of Adnexal Masses states “Repeat imaging is recommended if there is uncertainty regarding a diagnosis …. The frequency of repeat imaging has not been determined” (31). Although the optimal interval between follow-up studies for stable masses has not been rigorously studied, reimaging stable masses at 3-month intervals has been adopted by many as a reasonable schedule (26, 28, 29, 32). In our study of postmenopausal women with small complex masses, all five cancers diagnosed during follow-up demonstrated growth on the first repeat ultrasound, done 2–7 months later (13). All patients who had reimaging done within 6 months were found to have stage I disease at surgery. These results support the view that 3-month intervals between exams provide an opportunity to detect worrisome growth while still supporting early detection. If progression of the mass is seen on repeat imaging, surgical removal is appropriate. In our experience, women also elect eventual surgery due to cumulative anxiety or because a follow-up ultrasound raises concerns for progression even though the mass is unchanged, due to variability in ultrasound technique and reporting styles. Therefore, if monitoring is to be effective, follow-up studies should state explicitly whether any changes observed are potentially due to variation in image acquisition, in order to differentiate masses that are equivocably changed from those that are definitely changed.

## Duration of Monitoring for Stable Masses

The question of how long monitoring should be continued for stable but persistent masses is best viewed from the standpoint of potential benefit versus potential risk. Since the only potential benefit of monitoring asymptomatic masses is to identify masses that are malignant by observing growth over time, the longer a mass is observed to be stable, the less likely it is to represent a malignancy, and therefore the lower the potential benefit of further monitoring. Within the population-based cohort we studied, all five epithelial cancers as well as nine borderline tumors demonstrated clear growth on their first follow-up ultrasound (13). Similarly, in the Kentucky study, all malignant tumors were identified as worrisome within a relatively short time frame from initial detection, with malignant tumors receiving only 2.1 scans over a mean 2.3 months prior to removal (21). The recognition that ovarian cancers are heterogeneous in behavior with some tumors having more indolent growth patterns than others has led to a new paradigm that categorizes ovarian cancers as Type 1 or Type 2 based on their purported pathogenesis (33, 34). Type 2 cancers, which include high grade serous histology and represent the majority of ovarian epithelial malignancies, are thought to arise primarily from fallopian tube rather than ovarian precursors, which helps explain the failure of screening trials to detect these cancers at early stage. Type 1 cancers, which include low grade endometrioid, clear cell, and mucinous histologies, are thought to arise from endometriosis or ovarian precursors and generally demonstrate a more indolent growth pattern. Therefore, the paradigm raises the question of whether screening, or indefinitely prolonged monitoring of stable masses, which eventually becomes tantamount to screening, confers significant benefit for early detection of Type 1 cancers. This is an open question. However, any prediction of benefit from detection of Type 1 cancers must take into consideration the fact that benefit is realized only if the stage at diagnosis is earlier than would otherwise occur. Since this subset of cancers come to clinical attention much more often at early stage (35), such benefit is less likely. In our study, three of the seven cancers were Type 1 and all demonstrated growth on follow-up ultrasound within 7 months with no additional cancer diagnoses within 24 months of follow-up (13). Similarly, in UKCTOCS, all of the Type 1 cancers found among women who demonstrated an abnormality on initial ultrasound evaluation were diagnosed within the first year of follow-up (9). No measurable benefit from monitoring of stable masses beyond 2 years has ever been demonstrated.

## Potential Harms of Monitoring

Although the potential benefit of monitoring wanes over time, the potential harms are cumulative. The most significant harm occurs from unnecessary surgery for a benign asymptomatic mass. Benign adnexal masses are known to be extremely common. Depending on the size threshold of what constitutes a “mass,” autopsy studies have shown that between 17 and 56% of postmenopausal women who died from non-gynecologic causes harbor ovarian cystic or solid masses at the time of death (36, 37). Although surgical removal is appropriate for symptomatic masses, there is no clear benefit of removal of a benign asymptomatic adnexal mass. Thus, surgery that is done for an asymptomatic mass that does not reveal cancer is appropriately considered as a harm in cancer screening trials. Although minimally invasive techniques have lowered overall morbidity of surgery, such procedures were still found to be associated with an average 6% serious complication rate across screening trials (11). If bilateral salpingo-oophorectomy is done, depending on patient age, there is also potential harm from loss of hormone function, as negative impacts on cardiovascular health, bone health, and possibly cognitive function have been reported in women whose ovaries were removed prior to 50 years of age (38). Costs to the health-care system from surgery and complications as well as both direct and indirect costs to patients are substantial. Although initial monitoring helps to avoid immediate surgery, prolonged monitoring of stable masses increases the likelihood of unnecessary surgery for incidental findings. It is not uncommon for a woman who is being followed for a stable adnexal abnormality to be found on repeat imaging to have a new adnexal abnormality, given the high prevalence of adnexal lesions, which then triggers another round of evaluation with either surgery or observation.

## Conclusion

In summary, ultrasound monitoring of adnexal masses is valuable in identifying early cancers among women who have small masses are asymptomatic and do not demonstrate other signs of cancer such as elevated CA125 or ascites. However, the overall risk of cancer for these women is very low. A short-term repeat ultrasound at 6–8 weeks to evaluate for either regression or growth helps to avoids surgery on transient masses and does not appear to worsen prognosis in the event that the mass represents an early cancer. In this population, the ultrasound criteria used to label adnexal masses as “highly worrisome,” and therefore excluded from consideration of any monitoring, should be clearly defined and relatively stringent, given the overall low risk of malignancy. The presence of significant solid components that demonstrate vascular flow appears to be the ultrasound characteristic for which there is the greatest consensus as to its specificity for malignancy. Masses demonstrating clear progression during monitoring should be removed. For stable masses, repeat ultrasound at 3-month intervals, to observe for worrisome growth or changes in complexity is appropriate. However, since the potential benefit in terms of cancer identification wanes with time, the duration of monitoring of stable masses should be limited to 1–2 years in order to limit potential harms from overtreatment and overdiagnosis.

## Author Contributions

ES-B: literature review and manuscript writing. WK: literature review and manuscript editing.

## Conflict of Interest Statement

The authors declare that the research was conducted in the absence of any commercial or financial relationships that could be construed as a potential conflict of interest.
